# Enzyme Stability in Nanoparticle Preparations Part 1: Bovine Serum Albumin Improves Enzyme Function

**DOI:** 10.3390/molecules25204593

**Published:** 2020-10-09

**Authors:** Jason Thomas Duskey, Federica da Ros, Ilaria Ottonelli, Barbara Zambelli, Maria Angela Vandelli, Giovanni Tosi, Barbara Ruozi

**Affiliations:** 1 Nanotech Lab, Te.Far.T.I., Department Life Sciences, University of Modena and Reggio Emilia, 41125 Modena, Italy; jasonthomas.duskey@unimore.it (J.T.D.); federica.daros@unimore.it (F.d.R.); ilaria.ottonelli@unimore.it (I.O.); mariaangela.vandelli@unimore.it (M.A.V.); gtosi@unimore.it (G.T.); 2 Umberto Veronesi Foundation, 20122 Milan, Italy; 3 STEM Cell Lab, University of Milan, 20133 Milan, Italy; 4 Clinical and Experimental Medicine PhD Program, University of Modena and Reggio Emilia, 41125 Modena, Italy; 5 Laboratory of Bioinorganic Chemistry, Department of Pharmacy and Biotechnology, University of Bologna, 40127 Bologna, Italy; barbara.zambelli@unibo.it

**Keywords:** polymeric nanoparticles, enzyme delivery, enzyme stabilization, nanomedicine

## Abstract

Enzymes have gained attention for their role in numerous disease states, calling for research for their efficient delivery. Loading enzymes into polymeric nanoparticles to improve biodistribution, stability, and targeting in vivo has led the field with promising results, but these enzymes still suffer from a degradation effect during the formulation process that leads to lower kinetics and specific activity leading to a loss of therapeutic potential. Stabilizers, such as bovine serum albumin (BSA), can be beneficial, but the knowledge and understanding of their interaction with enzymes are not fully elucidated. To this end, the interaction of BSA with a model enzyme B-Glu, part of the hydrolase class and linked to Gaucher disease, was analyzed. To quantify the natural interaction of beta-glucosidase (B-Glu,) and BSA in solution, isothermal titration calorimetry (ITC) analysis was performed. Afterwards, polymeric nanoparticles encapsulating these complexes were fully characterized, and the encapsulation efficiency, activity of the encapsulated enzyme, and release kinetics of the enzyme were compared. ITC results showed that a natural binding of 1:1 was seen between B-Glu and BSA. Complex concentrations did not affect nanoparticle characteristics which maintained a size between 250 and 350 nm, but increased loading capacity (from 6% to 30%), enzyme activity, and extended-release kinetics (from less than one day to six days) were observed for particles containing higher B-Glu:BSA ratios. These results highlight the importance of understanding enzyme:stabilizer interactions in various nanoparticle systems to improve not only enzyme activity but also biodistribution and release kinetics for improved therapeutic effects. These results will be critical to fully characterize and compare the effect of stabilizers, such as BSA with other, more relevant therapeutic enzymes for central nervous system (CNS) disease treatments.

## 1. Introduction

Numerous proteins and peptides have received worldwide approval as therapeutic agents from regulatory authorities, and several hundred more are being tested in clinical trials. Among therapeutic proteins, enzymes represent a small but rapidly growing market due to their potential application in curing important, rare, and deadly diseases. For example, enzyme replacement therapy (ERT) is undoubtedly the most promising therapeutic approach for mucopolysaccharidosis (MPSs), as well as for some other forms of lysosomal storage disorders (LSDs) such as Gaucher disease [1], Fabry disease [2], and Pompe disease [3], in which remarkable clinical benefits are currently obtained [4]. Unfortunately, clinical applications of these macromolecules are hampered by numerous obstacles to their successful delivery and targeting. Enzymes frequently exhibit a rapid decrease in enzyme kinetics and specific activity due to their destabilization and short half-lives in serum, requiring frequent administration to maintain therapeutic levels. Also, improving their biodistribution remains a striking challenge as there is often poor accumulation in the pathological sites (especially in the central nervous system (CNS), bone, cartilage, cornea, and heart) [5,6]. A possible solution to these limitations lies on tailored delivery of enzymes by means of biodegradable and biocompatible nanomedicines (NMeds). Different NMeds, including polymeric micelles, liposomes, and polymer- and lipid-based nanoparticles (NPs) [7] have been exploited for enzyme encapsulation as they are able to protect enzymes from undesired immunologic reactions and biodegradation, to ameliorate the biodistribution of the enzyme, to improve the pharmacological response, and to modulate enzyme release at the target site limiting undesirable side-effects [8,9]. In this field, polymeric NPs, particularly those made of polylactide-co-glycolic acid (PLGA), have attracted considerable interest over the last few years as versatile tools for enzymatic delivery [10,11,12]. While many promising results have been described, frequently authors declare a certain criticism related to the formulation aspect of enzyme-loaded NP formulations due to both low loading efficiency and the maintenance of the enzymatic activity [13,14].

Generally, in the function of the physico-chemical properties of the enzyme and the characteristics of the desired PLGA NPs, several techniques including nanoprecipitation, emulsion, sonication, extrusion and other stressful mechanical and chemical processes are employed during the preparation process. In particular, double emulsion water-oil-water protocols (also known as w/o/w techniques) have been widely tested for the encapsulation of hydrophilic molecules, such as therapeutic proteins and enzymes. This technique imposes substantial stresses on the chemical integrity and the native three-dimensional structure of proteins [15,16]. For example, protein inactivation and aggregation at the water/organic interface, probably due to interfacial adsorption followed by protein unfolding and aggregation, is one of the most detrimental events when applying this technique precluding the use of NPs [17]. Similarly, the application of ultrasonic energy to obtain nano-emulsions is important to consider as a critical process parameter. The complex physical and chemical phenomena that occur during the cavitation process (with extreme localized temperatures and pressures generated) can alter the molecular structure and cause potential surface charge changes in the enzyme [18].

Another critical methodology that significantly stresses the enzyme includes the freeze-drying process (such as lyophilization), applied to concentrate the enzyme and to allow stable storage of the NPs over time as was observed in our preliminary studies and confirmed in other literature sources [5,19].

To limit or solve the problems of enzyme encapsulation into polymeric NPs, the addition of a stabilizer such as polyols, sugars, inorganic salts, surfactants, and polymers [20,21] to protect enzymes by masking them from denaturation and to prevent protein inactivation during process manipulation have been reported [16]. Another approach to stabilize enzymes against emulsification stress is based on the addition of bovine serum albumin (BSA). The high solubility of albumin (up to 40% *w*/*v*) at pH 7.4, its stability at pH values of 4 to 9, and temperature variations (up to 60 °C when heated for 10 h) without any deleterious effects make it an attractive macromolecular stabilizer [22,23,24,25]. As an example, Chang and co-workers demonstrated that BSA was able to stabilize an enzyme by promoting hydrophobic interactions and increasing the viscosity of the enzyme solution [26]; however, this must be carefully balanced as increasing the protein concentration and viscosity increases the probability of aggregation which could lead to decreased enzyme availability or non-uniform NP formation [27].

Considering the need for a better understanding of the protective effect of BSA on enzymes, we rationally studied the complexation between beta-glucosidase (B-GLU), a model enzyme that falls in the hydrolase enzyme class and is linked to the LSD Gaucher, with a stabilizer (BSA), combining this strategy with a modulate delivery through polymeric nanoparticles (PLGA NPs) to optimize the formulation strategy aiming to stabilize an enzyme, preserve its activity, and modulate release at the target site. To this aim, we characterized the interaction between B-GLU and BSA in solution using microscopy and ITC analysis to discover the interaction characteristics of the BSA stabilizer and the enzyme. We then evaluated the effect of using stabilized BSA:enzyme complexes in nanoparticle formation by means of a full chemico-physical characterization (charge, size, enzyme loading, activity, and release), followed by enzyme activity and release studies to establish if the addition of BSA:stabilizer complexes to a formulation would lead to more therapeutically relevant enzyme-based nanomedicines. 

## 2. Results

### 2.1. BSA/B-GLU Interaction

To better understand how BSA promotes the stabilization effects on B-GLU in aqueous solutions, titration of B-GLU with BSA was studied through isothermal titration calorimetry (ITC).

**Figure 1:**
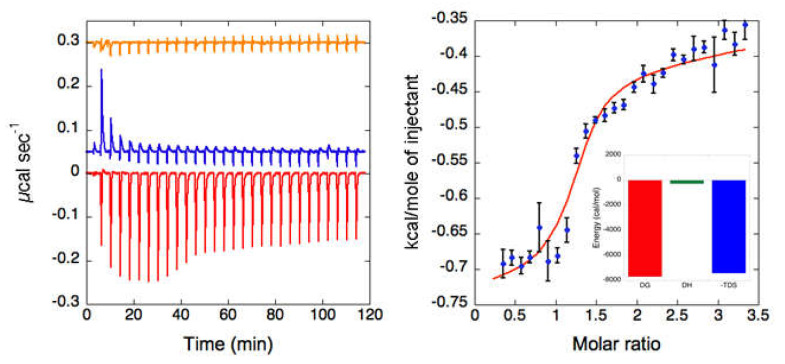
Isothermal titration calorimetry (ITC) analysis of the bovine serum albumin: beta-glucosidase (BSA:B-GLU) complex: (**Left**) Raw titration data of BSA titrated over B-GLU in PBS buffer pH 6 (red trace), BSA titrated over PBS buffer pH 6 (blue trace) and of PBS buffer pH 6 titrated over B-GLU (66 µM, orange trace). (**Right**) Binding isotherm of BSA titration over B-GLU obtained by integrating raw data for the protein titration and subtracting the corresponding control. The blue dots represent the experimental data, and the red curve represents the fit of the data using an independent sites approach using the software Affinimeter. In the inset, the thermodynamic signature is reported.

The interaction of B-GLU with BSA is an exothermic reaction, as indicated by negative peaks following each addition of BSA into the enzyme solution (Figure 1 left). The heat of the B-GLU dilution was negligible (Figure 1 left , orange curve), while a minor endothermic heat of dilution for BSA was visible for the first two additions (Figure 1 Left, blue curve), which were discarded from the data analysis. Integrated heat data (Figure 1 right) showed a single inflexion point, which likely indicated a single binding event. Accordingly, the data were fitted (GoF = 50.81%) using a scheme involving a single binding site (*N* = 1.23 ± 0.01), providing an affinity constant *K_A_* = (5.3 ± 0.9) × 10^5^ M^−1^ (*K_D_* = 1.9 ± 0.3 μM). The reaction is entropically driven with very small enthalpic contribution and favourable entropic values (ΔH = −0.26 ± 0.09 kcal mol^−1^ and ΔS = +25.4 cal mol^−1^ K^−1^). In particular, the stoichiometry of the complex between BSA and B-GLU is characterized by a molar ratio close to 1:1.

To further characterize the complex formation, solutions of different BSA:B-GLU molar ratios ranging from 2:1 to 40:1 (high excess of surfactant) were prepared and analyzed (concentration of B-GLU 10 mg/mL to simulate conditions of the aqueous phase during formulation). The excess of BSA in respect to the enzyme molarity was necessary to assure at least the partial saturation of the B-GLU:BSA binding at the nM concentrations used for these experiments, which were distant from the value of the dissociation constant measured by ITC. Photon Correlation Spectroscopy (PCS) was applied to 2:1 (Complex **1**), 10:1 (Complex **2**), 20:1 (Complex **3**) BSA:B-GLU molar ratio (Table 1). Unfortunately, the high viscosity of 3 nM BSA solution, used to obtain 40:1 (Complex **4**) BSA:B-GLU molar ratio hindered reliable results by this method. The size distribution of free B-GLU shows the presence of a predominant peak (80% of the sample) of about 10 nm, most likely representing the globular form of the enzyme and minor amounts of larger structures with a diameter > 100 nm. BSA solutions corresponding to the concentrations used in the complexations (0.15, 0.75, 1.5, and 3 nM; Complexes **1**–**4**, respectively) were also tested in the absence of the enzyme, showing a reproducible bimodal distribution with a predominant component of about 2–3 nm (60% of samples) and a secondary peak of about 20–38 nm (30–40% of the sample), probably due to aggregation. This finding is in line with other data present in literature, which showed differences in size depending on pH values: in particular, a primary peak, with a particle diameter ranging from 2 to 4 nm was found in solutions with pH 4–9 (compact spheroid particle), while the diameter increased at pH < 4 (extended form) [28,29]. The co-presence of BSA and B-GLU created a more complex dimensional distribution, with a significant increase of polydispersity; in particular, the 3 nm fraction decreased proportionally with an increased molar ratio of BSA to B-GLU (from 50% to 16%), corresponding to the increased contribution of larger structures (diameter > 50 nm) suggesting the re-organization of proteins in solution. However, a noteworthy observation, was that the activity of the enzyme appeared to be unaffected by the increased presence of BSA (~16,000 pmol product/ug B-GLU). Unfortunately, the high viscosity of a 3 nM BSA solution hindered reliable results by this method.

**Figure 2:**
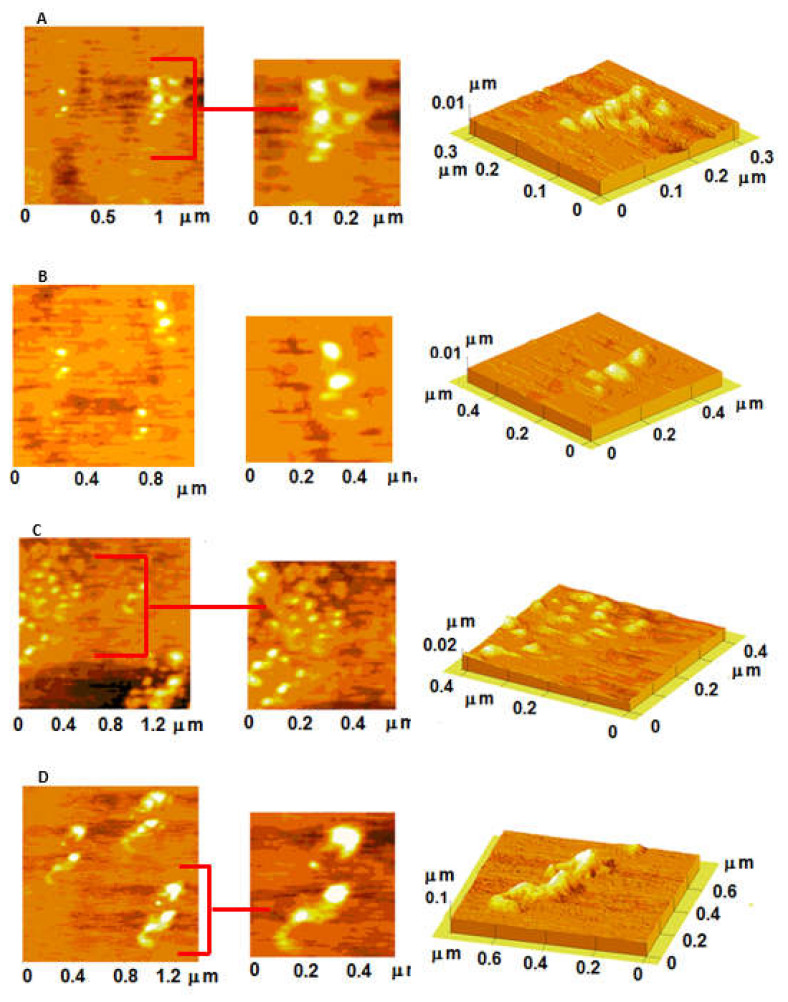
Atomic force microscopy (AFM) images of BSA:B-GLU complexes: 2:1 (panel **A**), 10:1 (panel **B**), 20:1 (panel **C**) and 40:1 (panel **D**).

The structure of all enzyme:BSA solutions were analyzed using atomic force microscopy (AFM). When the molar ratio between BSA and B-GLU was lower than 20:1 very small structures (diameter of about 20 nm) with a slightly collapsed spherical shape were observed on the mica substrate (Figure 2A–C). On the contrary, the mixtures of BSA:B-GLU 40:1 molar ratio gave the formation of irregular and heterogeneous structures, with large amounts of background signal (Figure 2D).

### 2.2. NPs Loaded with BSA:B-GLU Complexes

B-GLU complexed with BSA, along with control samples, were formulated in PLGA NPs by means of a double emulsion (w/o/w) process. The chemical-physical properties of control samples (empty NPs, NPs formulated in BSA solution without B-GLU, and NPs loaded with B-GLU without BSA), and NPs loaded with BSA:B-GLU complexes are reported in Table 2. Control NPs without enzyme-containing BSA at concentrations of 0.15, 0.75, 1.5, and 3 nM (corresponding to the concentrations of complexes **1**–**4**, respectively) exhibited homogenous NPs with an increase in size from 185 to 265 nm with increasing concentrations of BSA, a stable negative surface charge (around −20 mV) and a PDI always ~ 0.02 with the exception of the sample containing 3 nM BSA in which the PDI increased to 0.2, probably due to the higher viscosity of the suspension leading to variation in NP formation. Other control samples, namely B-Glu NPs prepared in the absence of BSA, formed 190 nm particles with a surface charge of −24 mV with good homogeneity as confirmed by small PDI values (0.017).

NPs loaded with BSA:B-GLU complex showed a slight increase of both the mean diameter (from 180 to 230 nm) and the heterogeneity of the population (from 0.06 to 0.15), with the exception of Complex **4** (40:1) which showed both polymodal and polydisperse population of structures. Zeta potential of all samples was unaffected.

**Figure 3:**
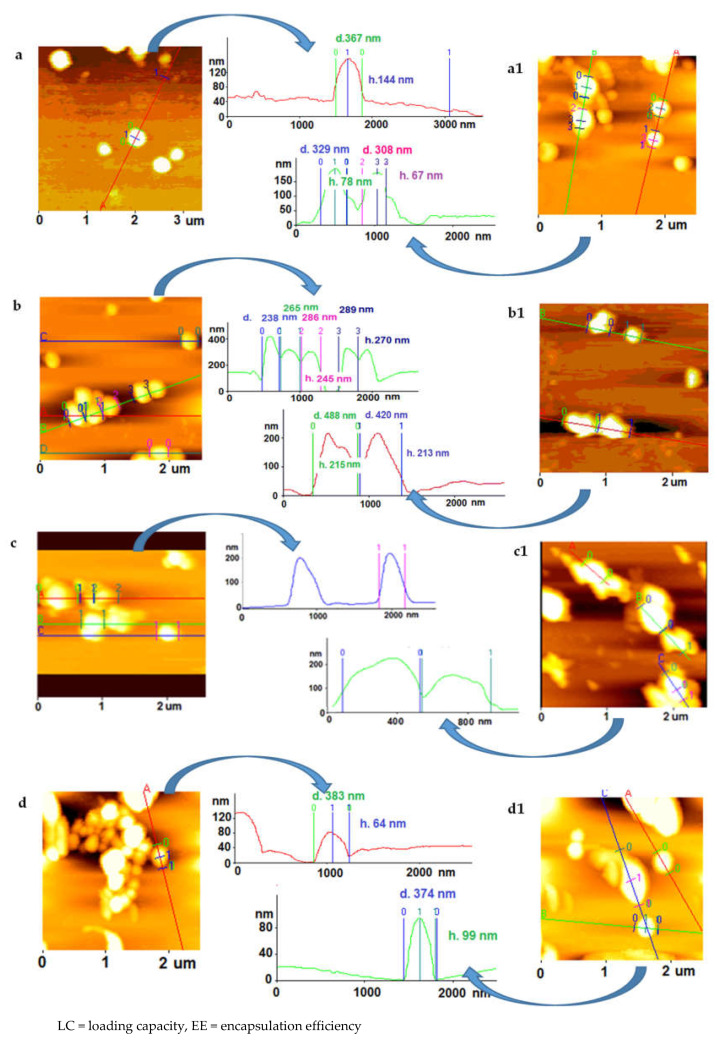
AFM images of the different NP preparations (**a**) NPs, (**a1**) NPs/B-GLU, (**b**) NPs/BSA 0,15 nM, (**b1**) NPs/Complex **1**, (**c**) NPs/BSA 0,75 nM, (**c1**) NPs/Complex **2**, (**d**) NPs/BSA 1,5 nM, (**d1**) NPs/Complex **3**. Note: All NPs containing BSA:enzyme complex were produced with 5 mg B-Glu. The control samples are highlighted in grey.

These values were supported by AFM measurements (Figure 3); NPs prepared in the presence of BSA (with or without B-GLU) tend to aggregate more on mica compared with PLGA NPs without stabilizer. Moreover, high concentrations of BSA in the formulation (BSA 3 nM and related NPs/Complex **4**) were highly viscous and strongly hampered the approach with the sample, leading to artefacts in the analysis. Similarly, the interaction between the sample and the substrate as well as the continuous movement of the tip on the sample can drag particles on the support [30]. When the analysis was possible, the diameters obtained by AFM image processing resulted in higher values compared with the PCS data and the heights of the NPs were not found to correlate with diameter: this finding was probably connected to the water evaporation entailed in the AFM sample preparation influencing the NP size. Taken together, the AFM analyses generally confirm the heterogeneity of samples with a diameter of NPs ranging from 200 to 1000 nm. 

Regarding pharmaceutical characterization, the presence of BSA:B-GLU complex in the NP preparations led to a strong variation on the percent yield of the NP recovery and encapsulation efficiency of the enzyme. In fact, the yield decreased proportionally with the increase of BSA concentration in the sample (from 80% to 30%). We hypothesized that this dramatic reduction in yield could be due to the relevant surfactant effect of both BSA and polyvinyl alcohol (PVA) on the suspension of NPs, leading to a less effective centrifugation outcome. In fact, the emulsifier action of BSA (in the inner aqueous phase) together with PVA (present in outer aqueous phase) could favour a reduction of both the dimension of the NPs and the interfacial tension leading to a very stable NPs suspension in the aqueous medium even after purification by centrifugation. 

Remarkably, the most relevant outcome connected to the BSA concentration variation used to stabilize B-GLU was the enzyme encapsulation efficiency (Table 2). Without BSA, only a poor amount of B-GLU was efficiently loaded into NPs, namely 0.6% considering loading capacity (LC) and close to 6% considering encapsulation efficiency (EE). Similar encapsulation values were recorded (LC close to 0.7% and EE close to 7%) when incorporating the complex formed using the conditions” defined by the optimal molar ratio between BSA:B-GLU as suggested by titration studies (2:1). Notably, when the concentration of BSA in the formed complex was increased above these values, particularly with a molar ratio BSA:B-GLU 20:1 (Complex **3**), encapsulation remarkably increased with LC 4% (4 mg B-GLU/100 mg of NPs) and EE 40%. 

A further increase in BSA (BSA:B-GLU 40:1 mom:mol) did not correspond to an increase in encapsulation (LC 1.2% and EE 11%), which could be related to possible aggregation or too high viscosity of solution leading to a loss in encapsulation efficiency.

### 2.3. B-Glu Release Study: Enzymatic Activity 

**Figure 4:**
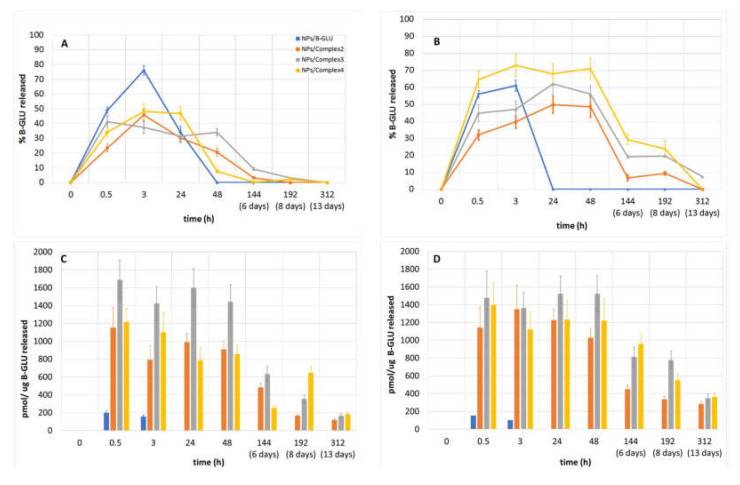
% of B-Glu released quantified by HPLC analysis at (**A**) pH 7.4 and (**B**) pH 4.5. The activity of enzyme/ug of B-Glu released from NPs at (**C**) pH 7.4 and (**D**) pH 4.5.

**Figure 5:**
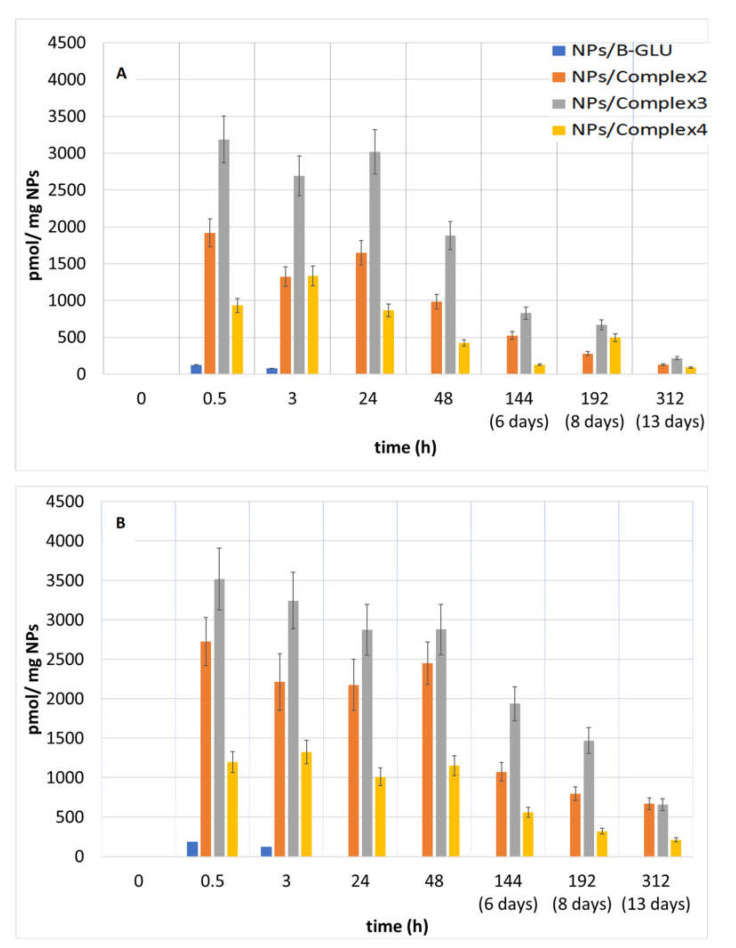
B-GLU activity per mg of NPs: (**A**) pH 7.4 and (**B**) pH 4.5.

The release of B-GLU from NPs was evaluated in buffered solutions considered biologically relevant: pH 7.4 to mimic the environment upon systemic administration in the blood plasma and pH 4.5 to mimic endocytotic intracellular trafficking once taken up by cells [31,32,33,34]. The analysis of enzyme release becomes complicated due to the variability not only in the amount of enzyme released but in the activity remaining of the enzyme once released. This is further complicated by the competitive actions of enzyme release/activity and enzyme degradation once in free solution over time. Therefore, to characterize the global effect, enzyme release was analyzed considering the percent and activity of enzyme released (Figure 4) was quantified by enzyme activity per mg of nanoparticles (Figure 5). 

The release of the enzyme from NPs at pH 7.4 and 4.5 was quantified by HPLC analysis (Figure 4A,B, respectively). In a pH 7.4 environment, B-GLU release from NPs without BSA as a stabilizer showed an almost complete burst release of ~80% over the first 3 h with no enzyme detectable at longer time points. On the contrary, NPs containing BSA:B-GLU complex exhibited a remarkably lower percentage of burst release with a slower maintained release of enzyme up until 48 h independent of which complex was used, and even as long as 144 h in the case of Complex **2** (Figure 4A).

More striking, the enzyme released from the NPs lacking BSA was almost completely inactive at both pH values tested (Figure 4C,D), even at short time points (300 pmol/ug B-GLU), where the majority of the enzyme was released by HPLC analysis, with no activity seen at longer time points suggesting complete enzyme degradation. NPs containing BSA:B-GLU complexes show significantly more activity than the enzyme released by NPs/B-GLU without BSA (at both pH). However, at short time points (3 h) the enzymatic activity (evaluated considering the pmol of product transformed by enzyme) of B-GLU released was between 5-fold (complexes **2** and **4**) and 8-fold (complex 3) higher than that of the enzyme released from NPs without BSA. Previous studies have shown that other LSD linked enzymes in a physiological pH solution, for 10 h at 37 °C, undergo destabilization with a loss of activity of about 40% [32,35,36]. This explains why in the experiments carried out both at pH 7.4 and 4.5 almost all enzyme amount can be quantified (80%) before degradation begins in the first 10 h and affects the kinetics and the complete release of BSA:B-GLU complex loaded into NPs over the 13 days of the experiment is never achieved as the degraded enzyme quantities were not quantifiable by HPLC. 

More importantly, the activity remained close to maximal levels out to 48 h both at pH 7.4 and 4.5. Even after 48 h, enzyme activity was still detectable and decreased steadily over 13 days, with a more rapid decline at pH 7.4 respective to pH 4.5. Preservation of enzyme activity is more remarkable when considering NPs/Complex **3**, which showed maximal observ with respect to ed encapsulation efficiency (EE 40%, Table 2). These two results indicate not only that the BSA:B-GLU complex is able to promote a prolonged release profile, but also stabilizes the enzyme in solution allowing a more therapeutically relevant activity profile over time.

When the enzyme activity was reported with respect to a weighed quantity of NPs (Figure 5A,B), the observed results showed similar kinetics and overall evaluations. In particular, maximal activity during the first 24 of the experiment, with a rapid decrease for NPs without BSA, and a slower decline for those containing the BSA-BGLU complex were observed.

## 3. Discussion

Delivery of therapeutic proteins has gained great interest for the treatment of a number of diseases such as lysosomal storage disorders. More specifically, enzyme delivery possesses extraordinary therapeutic potential if properly formulated due to the fact that by exploiting this approach, enzymes can lead to prolonged and stable effects. In fact, achieving enzyme stability in the blood and targeted delivery still remains a challenge and a limiting step in order to move forward to clinical applications. To this end, nanoformulations could be reasonably considered as a resolutive strategy as it can achieve both specific targeting to disease sites and afford pharmaceutical advantages such as proper enzyme stabilization to protect pharmacological activity. Since BSA was shown to have a protective effect on enzymes, in this work, we exploited that effect to better characterize and understand the protective effect of complexes between BSA and B-GLU. The complex formation between BSA and B-GLU was found to be an exothermal reaction with a 1:1 molar ratio and an affinity in the µM range. When formulated into PLGA NPs, the very low concentrations of the enzyme needed for the encapsulation required an excess of BSA for complex formation. Results clearly indicated that a BSA:B-GLU ratio of 10:1 is the most efficient to achieve good performances in terms of encapsulation efficiency. An increasing concentration of BSA to 20:1 molar ratio was also considered: while it, unfortunately, led to a decreased recovery yield, it positively affected the encapsulation by dramatically increasing enzyme loading per mg of NPs. On the other hand, a further increase of BSA concentration (BSA:B-GLU 40:1) did not correspond to an increase in encapsulation, likely due to aggregation or too high viscosity of the solution that prevented an optimal condition for the reaction to occur.

Most importantly, the BSA:B-GLU complex stabilized the enzyme, slowing down its release from the NPs and extending its quantifiable activity up to more than ten days in a physiologically relevant environment.

In conclusion, a proper design of a strategy based on the optimization of enzymes stabilization could have dramatic effects in the field by increasing enzyme delivery as well as creating more stable, potentially long-term treatments. This will lead to a better understanding and the ability to compare various therapeutic enzymes for an increased therapeutic potential by limiting dose requirements, toxicity and off-target effects in order to cure previously untreatable diseases.

## 4. Materials and Methods

### 4.1. Materials

Poly(d,l-lactide-co-glycolide) (PLGA,RG503H,MW ≅ 11,000) was used as received from the manufacturer (Boehringer-Ingelheim, Ingelheim am Rhein, Germany). Bovine Serum Albumin (BSA, 66 KDa), B-Glucosidase (B-GLU, MW 135 KDa) and polyvinyl alcohol (PVA, MW 15,000) were purchased from (Sigma-Aldrich, Milan, Italy). A MilliQ water system (Millipore, Bedford, MA, USA), supplied with distilled water, provided high-purity water (18 MO). All other chemicals were of analytical grade.

### 4.2. BSA:B-GLU Complexes

BSA:B-GLU complexes were prepared by adding B-GLU (5 mg) into a BSA solution at different concentrations (0.15, 0.75, 1.5, and 3 nM) in 500 uL MilliQ water.

### 4.3. NPs Preparation

NPs were obtained by a double emulsion method (w_1_/o/w_2_), adopted to increase the loading of hydrophilic molecules [37,38,39]. Briefly, 0.5 mL B glucosidase (5 mg) in an aqueous solution with or without BSA (0.15, 0.75, 1.5 and 3 nM) was emulsified in polymer solution (50 mg of PLGA in 2.5 mL CH2Cl2) under cooling (5 °C) by using a probe sonicator (MicrosonUltrasonic cell disruptor, Misonix Inc. Farmingdale, NY, USA) at 80 W for 45 s to obtain a w/o emulsion (first inner emulsion). The first inner emulsion was rapidly added to 12 mL of 1% (w:v) PVA aqueous solution and the w/o/w emulsion was formed under sonication (80 W for 45 s) at 5 °C. The formulation was mechanically stirred (1500 rpm) for at least 1 h (RW20DZM, Janke and Kunkel, IKA-Labortechnik, Staufen, Germany) at room temperature (RT) until complete evaporation of the organic solvent was achieved and finally purified by Hi-Speed Refrigerated Centrifugation (Beckman J21, Beckman Coulter, Indianapolis, IN, USA) at 16,000 rpm for 10 min at 5 °C. The supernatant was discarded, and the NPs were washed several times and re-suspended in 4 mL Milliq water. From the NP resuspension, 1 mL was lyophilized and weighed for a percent yield recovery (see below).

### 4.4. Characterization of BSA:B-GLU Complexes and NPs

#### 4.4.1. ITC Titration (Protein: Enzyme Interaction)

An ITC titration of the BSA and B-GLU interaction was evaluated at 25 °C using a high-sensitivity VP-isothermal titration calorimetry (ITC) microcalorimeter (Malvern Panalytical, Malvern, UK). The reference cell was filled with deionized water. The B-GLU solution was prepared by re-suspending 50 mg of protein powder in the reaction buffer (PBS, pH 6). A BSA stock solution at 25% *w*/*v* concentration was passed on a PD SpinTrap G-25 (Ge Healthcare) pre-equilibrated with reaction buffer. The protein was eluted at a concentration of 13% *w*/*v* and subsequently diluted to 1 mM with the reaction buffer to load the ITC syringe. Each experiment started with a small injection of 1–2 μL of BSA water solution, which was discarded from the analysis of the integrated data in order to avoid artefacts due to diffusion through the injection port occurring during the long equilibration period locally affecting the protein concentration near the syringe needle tip. The first addition was added only after baseline stability had been achieved. In each individual titration, 10 μL of 1 mM BSA solution was injected into a solution of 66 μM B-GLU using a computer-controlled 310-μL microsyringe. To allow the system to reach equilibrium, a 300 s delay was applied between each ligand injection. Control experiments obtained by titrating BSA into the reaction buffer or by titrating the reaction buffer into a B-GLU solution were performed. Integrated heat data obtained for each titration were fitted using a nonlinear least-squares minimization algorithm to a theoretical titration curve, using AFFINImeter (https://www.affinimeter.com/app/index.php/auth/login), using the independent sites approach. *N* (stoichiometry), ΔH (reaction enthalpy change, cal mol^−1^) and *K_A_* (binding constant, M^−1^) were the thermodynamic fitting parameters. The parameter Q_dil_ (heat of dilution, cal mol^−1^) was also adjusted as fitting parameters. The reaction entropy was calculated using the relationships ΔG = −RTlnK_A_ (R = 1.9872 cal mol^−1^ K^−1^, T = 298 K) and ΔG = ΔH − TΔS. The reliability of the obtained fits was evaluated using the Goodness of Fit (GoF) parameter provided by the software.

#### 4.4.2. AFM Analyses

Morphology of both BSA:B-GLU Complexes and NPs were evaluated by means of atomic force microscope (AFM, Park Instruments, Sunnyvale, CA, USA) analysis at RT. (about 25 °C) operating in air and in non-contact mode using triangular silicon tips. The resonant frequencies of the cantilever were found to be about 160 kHz. Before the analysis, a drop (20 μL) of the complexes or water-diluted NP suspensions (about 0.01 mg/mL) were applied to a small mica disk (1 cm × 1 cm); after 2 min, the excess water was removed using a paper filter. The topographical images obtained, also called “height” images, were flattened using second-order fitting to remove sample tilt.

#### 4.4.3. Photon Correlation Spectroscopy (PCS) Analyses

The Mean particle size (Z-Average) and polydispersity index (PDI) of all samples (BSA:B-GLU Complexes and NPs) were determined at 25 °C by PCS using a Zetasizer Nano ZS (Malvern, UK; Laser 4 mW He–Ne, 633 nm, Laser attenuator Automatic, transmission 100–0.0003%, Detector Avalanche photodiode, Q.E. > 50% at 633 nm, T = 25 °C). The results were normalized with respect to a polystyrene standard suspension. The zeta potential (ζ-pot) was measured by using the same equipment with a combination of laser Doppler velocimetry and phase analysis light scattering (PALS). All the data are expressed as means of at least three individual preparation lots measured in triplicate.

#### 4.4.4. Yield

A defined amount of purified NPs (around 10 mg) was freeze-dried (−60 °C, 1 × 10^−3^ mm/Hg for 48 h; LyoLab 3000, Heto-Holten, Allerod, Denmark) and the yield (Yield%) was calculated as follows:

Yield (%) = ((mg of freeze-dried sample)/(mg PLGA + mg of enzyme used for preparation)) × 100(1)

### 4.5. Quantification of Loaded Enzyme and Enzymatic Activity

#### 4.5.1. Enzyme Entrapment Efficiency (EE) and Loading Capacity (LC)

To quantify the amount of enzyme-Glu loaded into the NPs, an exact amount of freeze-dried loaded NPs (10 mg) was dissolved in DCM (1 mL). Then, MilliQ water (2 mL) (in which enzyme, but not PLGA, is soluble) was added. The mixture was electromagnetic stirring for at least an hour to allow complete evaporation of the organic solvent and polymer precipitation. The suspension was filtered (acetate cellulose filters, porosity 0.20 μm, Sartorius), and the amount of B-GLU in aqueous solution was quantified by analyzing 50 µL of the solution by RP-HPLC. The HPLC apparatus (JASCO Europe, Cremella, Italy) comprised a Model PU-2089 Plus pump provided with an injection valve with a 50 µL sample loop (Jasco, Model 7725i) with a C_8_ analytical column (Aeris™ 3.6 µm WIDEPORE XB-C8 200 Å, Phenomenex, Bologna, Italy). Solutions and mobile phases were freshly prepared before each. Elution was obtained using a gradient consisting of A [0.1% trifluoroacetic acid in MilliQ water (pH ~ 2)] and B (0.1% trifluoroacetic acid in acetonitrile) where the % of B was increased from 20–80 over 11 min at a flow rate of 1.2 mL/min. After the run was complete, 5 min was allowed at 20% B to re-equilibrate the column before the subsequent injection. All analyses were carried out under isothermal conditions at 70 °C (Column Heater, model 7971, Jones Chromatography). The eluent absorbance was monitored at 210 nm using a UV detector (Jasco UV-1575), and the chromatographic peak area was integrated and converted to a concentration of B-Glu based on a standard curve using the same methodology. Linearity of the standard calibration curve was achieved in the range of 30–400 µg/mL (y = 44391x – 22.476 R^2^ = 0.99).

The entrapment efficiency (EE) and the loading capacity (LC), expressed as a percentage, were calculated using the following formulas:

EE% = D/Td × 100(2)

LC% = D/W × 100(3)

where D is the amount of B-GLU loaded in the NPs, Td is the amount of B-GLU used for the preparation, and W is the weight of the NPs (polymer + enzyme).

All the data are expressed as the mean of at least three sample analyses.

#### 4.5.2. Quantification of Enzyme Activity

B-GLU activity was assayed by the microtiter plate method using the substrate 4-metilumbrelliferil-β-d-glucopiranoside. A reaction mixture of 100 uL containing 45 uL sample, 25 uL 4-metilumbrelliferil-β-d-glucopiranoside (2 mg/mL) substrate, and 25 uL Mc Ilvain buffer 4× pH 6 (monobasic phosphate 0.8 M, citric acid 0.4 M), and 5 uL water MilliQ was incubated under stirring (170 rpm) at 37 °C for 1 h. The reaction was stopped by adding 190 uL of glycine solution (250 mM, pH 10.7) to 10 ul of reaction solution; the product of the enzymatic reaction was monitored by using a fluorimeter, (Synergy HTX Multi-Mode Reader, BioTek, Winooski, VM, USA) with excitation lambda et 365 and the emission lambda at 488 nm. One unit of B-GLU activity was expressed as the amount of enzyme required to release one picomole of product under assay conditions and calculated based on a standard curve created on the same plate and same day by diluting a standard solution of 4-MU (5 uM) 0, 2, 5, 10, and 15 μL to a final volume of 200 in stop buffer.

Homogenate was diluted with millQ water to 2 mg/mL and sonicated, and GCase activity was determined in samples (20 μg protein) by hydrolysis of 5 mM 4-methylumbelliferyl-β-d-glucopyranoside in Mc IIvaine buffer (pH 5.4) in the presence of 22 mM sodium taurocholate at 37 °C for 1 h. The reaction was stopped by addition of 0.25 M glycine (pH 10.4), and 4-methylumbelliferone fluorescence was measured at 365 nm excitation, 450 nm emission [40,41]

### 4.6. Release Studies

Each sample (10 mg of lyophilized NPs) was re-suspended in an Eppendorf tube with 1 mL of buffer (PBS pH 7.4 or acetate buffer pH 4.5). The tube was closed and placed in a water bath (thermomix bu B. Braun, Milano, Italy) heated to 37 ± 0.1 °C, under stirring. At fixed time intervals (30 min, 3 h, 24 h, 48 h, 6 days, 8 days, or 13 days) samples were centrifuged (Spectrafuge 24D centrifuge, Edison, NJ USA at 13,300 rpm for 10 min to separate the NPs (pellet) from the released B-GLU (supernatant). The supernatant was filtered (cellulose acetate 0.20 μm), the precise volume was measured and divided into two aliquots which were processed by (1) HPLC analysis and (2) enzymatic activity as previously described.

## Figures and Tables

**Table 1:** Chemico-physical properties and activity of BSA:B-GLU complex.

	PDI ^a^ (S.D.)	Peak 1 nm ± S.D. (% ± S.D.)	Peak 2 nm ± S.D. (% ± S.D.)	Peak 3 nm ± S.D. (% ± S.D.)	Zpot ^b^ mV (S.D.)	pmol Product/ug B-GLU (Activity at pH 6.7 ± S.D.)
B-GLU	0.31 ± 0.04	9.5 ± 0.9 (78 ± 12)	144 ± 45 (24 ± 14)	/		16662 ± 800
BSA solution (0.15 nM)	0.28 ± 0.01	3.1± 0.1 (77 ± 3)	19 ± 3 (18 ± 2)	102 ± 25 (5 ± 2)	−20 ± 2	
BSA solution (0.75 nM)	0.33 ± 0.02	2.7 ± 0.4 (56 ± 9)	32 ± 7 (45 ± 11)	/	−19 ± 5	
BSA solution (1.5 nM)	0.38 ± 0.03	2.4 ± 0.4 (61 ± 12)	38 ± 3 (43 ± 7)	/	−13 ± 3	
BSA/B-GLU 2:1 mol mol	0.34 ± 0.01	3.5 ± 0.3 (46 ± 8)		288 ± 34 (53 ± 12)	−16 ± 1	16253 ± 775
BSA/B-GLU 10:1 mol mol	0.51 ± 0.04	2.5 ± 0.5 (25 ± 11)	62 ± 6 (48 ± 14)	241 ± 45 (25 ± 13)	−18 ± 4	16588 ± 675
BSA/B-GLU 20:1 mol mol	0.62 ± 0.05	2.3 ± 0.4 (16 ± 12)	73 ± 5 (42 ± 12)	369 ± 68 (41 ± 7)	−16 ± 3	17532 ± 943

^a^: Polydispersity index. ^b^: Zeta potential measurement of nanoparticle surface charge.

**Table 2:** Physico-chemical properties of nanoparticles (NPs).

Samples	Z-Average nm ± S.D.	PDI ^a^ ± S.D.	D(i)50 nm ± S.D.	D(i)90 nm ± S.D.	AFM Diameternm ± S.D.	ζ-pot mV ± S.D.	Yield% ^b^ ± S.D.	LC% ± S.D.	EE% ± S.D.
NPs	194 ± 17	0.06 ± 0.02	200 ± 16	311 ± 20	320 ± 47	−21 ± 3	86.2 ± 2.1		
NPs B-GLU	199 ± 28	0.17 ± 0.04	221 ± 26	376 ± 42	311 ± 69	−24 ± 6	77.6 ± 3.3	0.6 ± 0.1	5.7 ± 0.9
NPs/BSA (0.15 nM)	185 ± 11	0.07 ± 0.02	189 ± 24	267 ± 21	215 ± 77	−19 ± 3	72.2 ± 3.1		
NPs/Complex**1**	234 ± 19	0.21 ± 0.01	227 ± 12	365 ± 25	302 ± 34	−19 ± 2	69.2 ± 4.1	0.7 ± 0.3	6.8 ± 2
NPs/BSA (0.75 nM)	229 ± 21	0.09 ± 0.01	205 ± 20	297 ± 12	265 ± 67	−22 ± 3	61.2 ± 2.3		
NPs/Complex**2**	222 ± 17	0.11 ± 0.03	208 ± 19	332 ± 21	365 ± 76	−25 ± 3	59.1 ± 2.6	3.1 ± 1.9	31 ± 7
NPs/BSA (1.5 nM)	234 ± 14	0.10 ± 0.02	209 ± 11	318 ± 11	318 ± 51	−23 ± 3	56.9 ± 2.4		
NPs/Complex**3**	243 ± 31	0.14 ± 0.02	215 ± 11	339 ± 14	375 ± 64	−20 ± 3	51.1 ± 2.2	3.9 ± 1.4	38.7 ± 4
NPs/BSA (3 nm)	265 ± 67	0.27 ± 0.09	221 ± 12	401 ± 21	/	−23 ± 6	41.3 ± 3.1		
NPs/Complex**4**	266 ± 72	0.31 ± 0.09	253 ± 19	443 ± 16	/	−22 ± 7	33.6 ± 7.2	1.2 ± 0.4	11 ± 5

^a^: Polydispersity index. ^b^: Yield (%) = [(mg of freeze dried sample)/(mg PLGA + mg of enzyme used for preparation)] × 100.
